# Big data analysis of endovascular treatment for acute ischemic stroke: a study based on bibliometric analysis

**DOI:** 10.1055/s-0044-1789228

**Published:** 2024-08-31

**Authors:** Xin Lu, Huiying Che, Hongjian Guan

**Affiliations:** 1 Yanbian University Hospital, Department of Neurology, Yanji, Jilin Province, China.; 2 Yanbian University Hospital, Department of General Practice, Yanji, Jilin Province, China.

**Keywords:** Ischemic Stroke, Bibliometrics, Big Data, AVC Isquêmico, Bibliometria, Big Data

## Abstract

**Background** While bibliometric analyses are prevalent in the medical field, few have focused on ther endovascular treatment for acute ischemic stroke (AIS).

**Objective** To employ big data analysis to examine the research status, trends, and hotspots in endovascular treatment for AIS.

**Methods** We conducted a comprehensive search using the Web of Science (WOS) database to identify relevant articles on the endovascular treatment for AIS from 1980 to the present. We used various tools for data analysis, including an online platform ( https://bibliometric.com/app ), the Citespace software, the Vosviewer software, and the ArcMap software, version 10.8. A number of bibliometric indicators were collected and analyzed, such as publication date, country where the studies were conducted, institutions to which the authors were affiliated, authors, high-frequency keywords, cooperative relationship etc.

**Results** A total of 5,576 articles were retrieved. A substantial increase in the number of articles occurred after 2010. High-frequency keywords included terms such as *large vessel occlusion* , *reperfusion* , *outcome* , and *basilar artery occlusion* . Among the top 10 most productive authors, Raul G. Nogueira ranked first, with 136 published articles. Among the journals, *The New England Journal of Medicine* ranked first, with 5,631 citations. The United States has the closest collaborative ties with other nations.

**Conclusion** In the present study, we found that the reports of endovascular treatment for AIS gradually increased after 2010. Among them, Raul G. Nogueira was the most productive author in this field. *The New England Journal of Medicine* was the most cited, and it had the greatest impact. The Multicenter Randomized Clinical Trial of Endovascular Treatment of Acute Ischemic Stroke in the Netherlands (MR CLEAN) trial study was the most cited, and it was a landmark study. There are many interesting studies on endovascular treatment for AIS, such as ischemic penumbra, collateral circulation, bridging therapy etc.

## Resumo

**Antecedentes** Embora as análises bibliométricas sejam predominantes na área médica, poucas se concentraram no tratamento endovascular para acidente vascular cerebral isquêmico (AVCI) agudo.

**Objetivo** Empregar análise de big data para examinar o status da pesquisa, tendências e pontos críticos no tratamento endovascular para AVCI.

**Métodos** Realizamos uma pesquisa abrangente usando o banco de dados Web of Science (WOS) para identificar artigos relevantes sobre o tratamento endovascular para AVCI de 1980 até o presente. Usamos várias ferramentas para análise de dados, incluindo uma plataforma *on-line* ( https://bibliometric.com/app ), os *softwares* Citespace, Vosviewer e ArcMap, versão 10.8. Vários indicadores bibliométricos foram coletados e analisados, como data de publicação, país onde os estudos foram conduzidos, instituições às quais os autores eram afiliados, autores, palavras-chave de alta frequência, relacionamento cooperativo etc.

**Resultados** Um total de 5.576 artigos foram coletados. Um aumento substancial no número de artigos ocorreu após 2010. Palavras-chave de alta frequência incluíram termos como *oclusão de grandes vasos* , *reperfusão* , *desfecho* e *oclusão da artéria basilar* . Entre os dez autores mais produtivos, Raul G. Nogueira ficou em primeiro lugar, com 136 artigos publicados. Entre os periódicos, *The New England Journal of Medicine* ficou em primeiro lugar, com 5.631 citações. Os Estados Unidos têm os laços de colaboração mais próximos com outras nações.

**Conclusão** No presente estudo, descobrimos que os relatos de tratamento endovascular para AVCI aumentaram gradualmente após 2010. Entre eles, Raul G. Nogueira foi o autor mais produtivo neste campo. A revista *The New England Journal of Medicine* foi a mais citada e teve o maior impacto. O estudo clínico Multicenter Randomized Clinical Trial of Endovascular Treatment of Acute Ischemic Stroke in the Netherlands (MR CLEAN) foi o mais citado e foi um estudo de destaque. Existem muitos estudos interessantes sobre tratamento endovascular para AVCI, como penumbra isquêmica, circulação colateral, terapia de ponte etc.

## INTRODUCTION

The key to acute ischemic stroke (AIS) treatment lies in vascular recanalization, restoration of blood perfusion, and preservation of the reversible ischemic penumbra. Intravenous thrombolysis (IVT) has been established as an effective method; however, its effectiveness is relatively low when the AIS is caused by large vessel occlusion. 1 Moreover, IVT has a narrow time window and contraindications, prompting cerebrovascular physicians to explore the feasibility of endovascular treatment for AIS.

Early studies conducted in 2013 2 did not demonstrate superiority of endovascular treatment over traditional approaches. However, optimization of the endovascular treatment process continued, yielding more favorable outcomes. Encouragingly, the results of five multicenter randomized controlled trials published in 2015 1 3 4 5 6 provided substantial evidence supporting the advantages of endovascular therapy for AIS. As a result, these famous clinical trials have promoted the use of endovascular therapy for AIS.

Bibliometric analysis offers a systematic method to assess the landscape of scientific literature, revealing patterns in publication output, citation impact, author productivity, and collaboration networks across various research domains. While bibliometric analyses are prevalent in the medical field, few have focused on endovascular treatment for AIS. Thus, the present study aims to employ bibliometric analysis to uncover the research status and development trends in endovascular treatment for AIS. Through the current study, we anticipate that new insights into endovascular treatment for AIS can be gained, contributing to further advancements in the field.

## METHODS

**Figure 1:**
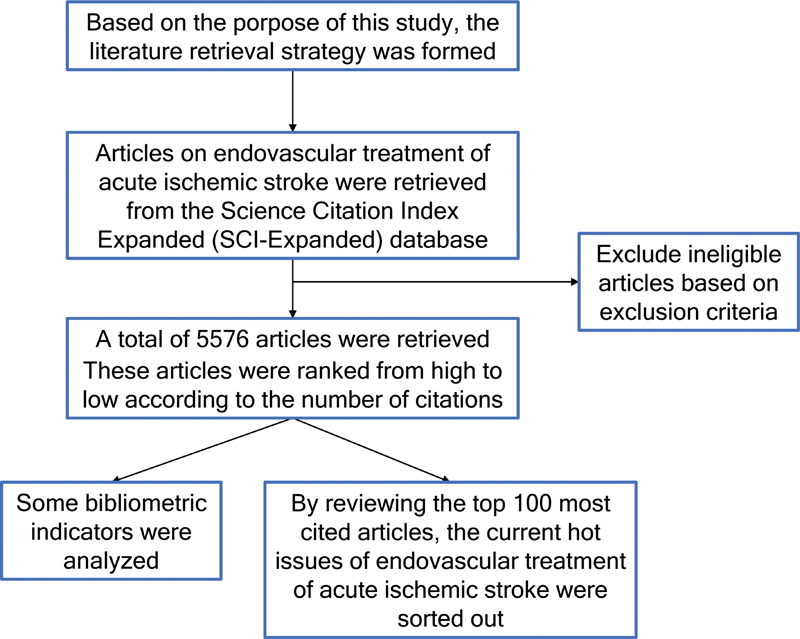
The flow chart of the present study.

The present study did not involve any human experimentation. Hence, ethical committee approval was not required. The flow chart illustrating the study design is presented in Figure 1 .

### Search strategy

We conducted a comprehensive search using the Web of Science (WOS) database to identify relevant articles on endovascular treatment for AIS from 1980 to the present. The search strategy is outlined in Table 1 . Articles were excluded based on the following criteria: lack of relevance to the research topic, language other than English, incomplete or missing data, and unavailability of the full text or abstract.

**Table 1:** The search strategy used in the study

Catalogue	Content
#1	(TI = (acute basilar artery occlusion)) OR TI = (acute ischemic stroke)) OR TI = (acute stroke)) OR TI = (alchemic stroke)) OR TI = (anterior cerebral artery occlusion)) OR TI = (basilar artery occlusion)) OR TI = (basilar occlusion)) OR TI = (carotid artery occlusion)) OR TI = (central cerebral artery occlusion)) OR TI = (cerebral artery occlusion)) OR TI = (cerebral infarct)) OR TI = (cerebral infarction)) OR TI = (cerebral ischemia)) OR TI = (infarction)) OR TI = (internal carotid artery occlusion)) OR TI = (internal carotid arteries occlusion)) OR TI = (internal carotid artery acute occlusion)) OR TI = (internal carotid artery obliteration)) OR TI = (internal carotid artery stenosis and occlusion)) OR TI = (ischemic stroke)) OR TI = (ischemic stroke*)) OR TI = (large vessel occlusion*)) OR TI = ( middle cerebral arterial obstruction)) OR TI = (middle cerebral artery embolism)) OR TI = (middle cerebral artery occlusion)
#2	(TI = (endovascular interventional treatment)) OR TI = (endovascular therapy)) OR TI = (endovascular thrombectomy)) OR TI = (endovascular treatment)) OR TI = (interventional therapy)) OR TI = (interventional treatment)) OR TI = (intra-arterial recanalization)) OR TI = (intra-arterial therapy)) OR TI = (intraarterial treatment)) OR TI = (intracranial stenting)) OR TI = (intravascular treatment)) OR TI = ( mechanical thrombectomy)) OR TI = (revascularization therapy)) OR TI = (flow restoration)) OR TI = (thrombectomy)
#3	#1 AND #2

Abbreviation: TI, Title.

### Data collection and analysis

We used various tools for data analysis, including an online platform ( https://bibliometric.com/app ), the Citespace software (free), the Vosviewer software (open source), and ArcMap 10.8 software (Environmental Systems Research Institute, Inc., Redlands, CA, United States).

**Figure 2:**
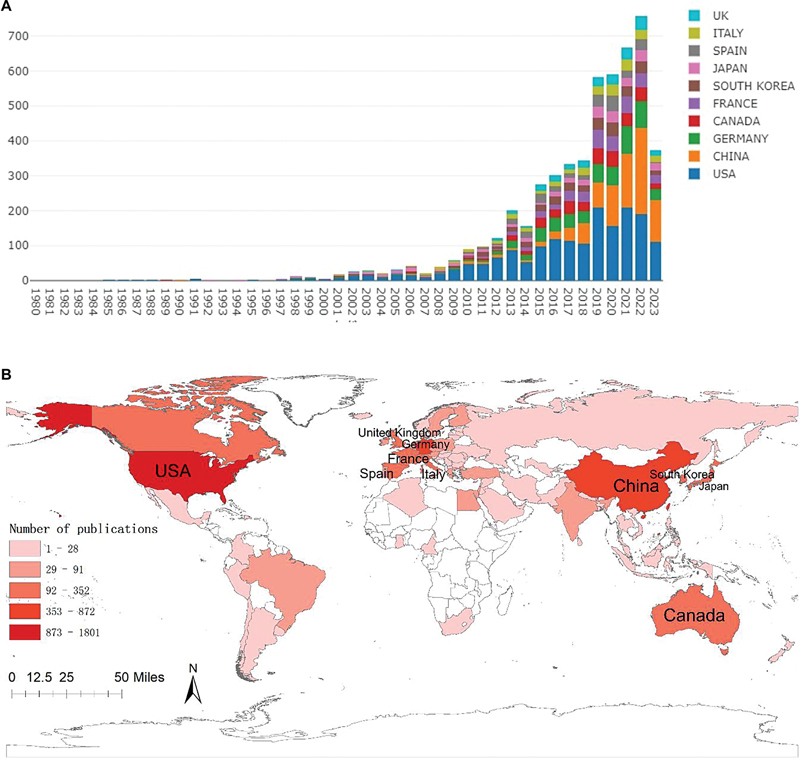
The number of articles published every year per country. ( **A** ) Publication date. ( **B** ) The countries with the highest number of published articles.

The study collected the following indicators ( Tables 2 3 4 5 , Figures 2 3 4 5 ):

**Table 2:** The top 10 most-cited articles

Rank	Year	Title	Citations
1	2015	A Randomized Trial of Intraarterial Treatment for Acute Ischemic Stroke 6	4,283
2	2016	Endovascular thrombectomy after large-vessel ischaemic stroke: a meta-analysis of individual patient data from five randomised trials 8	4,063
3	2015	Randomized Assessment of Rapid Endovascular Treatment of Ischemic Stroke 4	4,003
4	2015	Endovascular Therapy for Ischemic Stroke with Perfusion-Imaging Selection 5	3,804
5	2015	Thrombectomy within 8 Hours after Symptom Onset in Ischemic Stroke 3	3,237
6	2015	2015 American Heart Association/American Stroke Association Focused Update of the 2013 Guidelines for the Early Management of Patients With Acute Ischemic Stroke Regarding Endovascular Treatment A Guideline for Healthcare Professionals From the American Heart Association/American Stroke Association 45	1,444
7	2016	Time to Treatment With Endovascular Thrombectomy and Outcomes From Ischemic Stroke: A Meta-analysis 46	1,258
8	2013	A Trial of Imaging Selection and Endovascular Treatment for Ischemic Stroke 47	987
9	2012	Solitaire flow restoration device versus the Merci Retriever in patients with acute ischaemic stroke (SWIFT): a randomised, parallel-group, non-inferiority trial 48	978
10	2013	Endovascular Treatment for Acute Ischemic Stroke REPLY 49	955

**Table 3:** The top 10 most-cited institutions

Institution	Citations (n)	Number of articles	Annual number of citations
University of Calgary	8,357	362	23
University of California, Los Angeles	7,160	301	24
The University of Melbourne	6,146	156	39
University of Pittsburgh	4,140	142	29
University of Amsterdam	3,625	208	17
Bellvitge University Hospital (Hospital Universitari de Bellvitge)	3,446	17	203
Erasmus MC University Medical Center	3,003	84	36
Emory University	2,965	167	18
SUNY Buffalo	2,961	154	19
Maastricht University	2,889	158	18

**Table 4:** The top 10 most-cited journals

Journal	Citations (n)	Number of articles	Annual number of citations
*The New England Journal of Medicine*	5,631	46	122
*Stroke*	4,964	903	6
*Journal of Neurointerventional Surgery*	2,714	340	8
*The Lancet*	1,815	12	151
*American Journal of Neuroradiology*	1,194	116	10
*JAMA: The Journal of the American Medical Association*	803	36	22
*International Journal of Stroke*	584	431	1
*Journal of Stroke & Cerebrovascular Diseases*	503	173	3
*JAMA*	482	34	14
*Neurology*	427	255	2

**Table 5:** The top 10 most-productive authors

Author	Number of articles
Raul G. Nogueira	136
Mayank Goyal	132
Jeffrey L. Saver	112
Diederik W J Dippel	98
Aad van der Lugt	96
David S. Liebeskind	92
Charles B. L. M. Majoie	89
Benjamin Gory	84
Adnan I. Qureshi	84
Tudor G. Jovin	83

**Figure 3:**
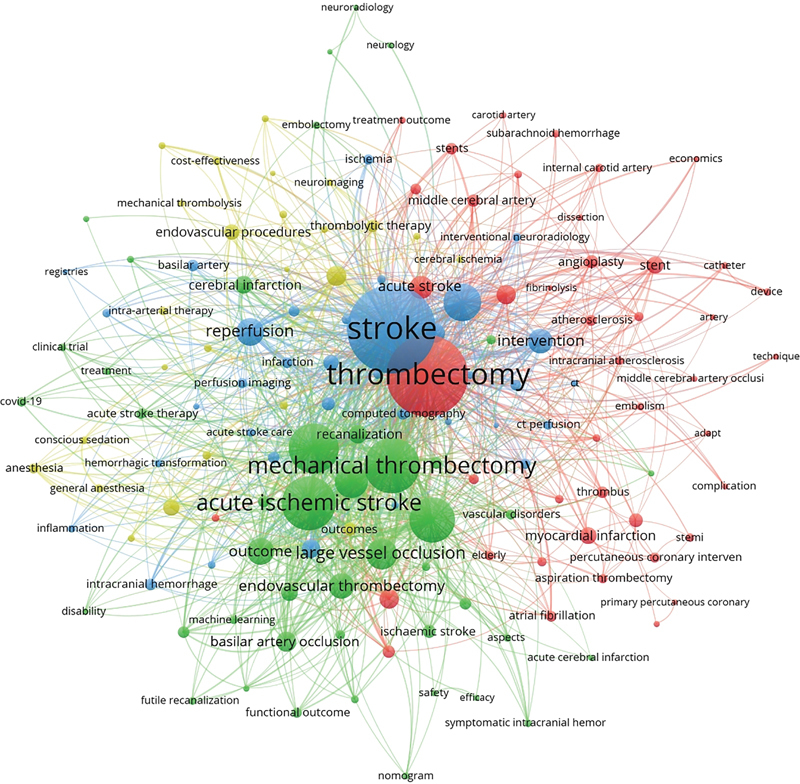
The network diagram of high-frequency keywords.

**Figure 4:**
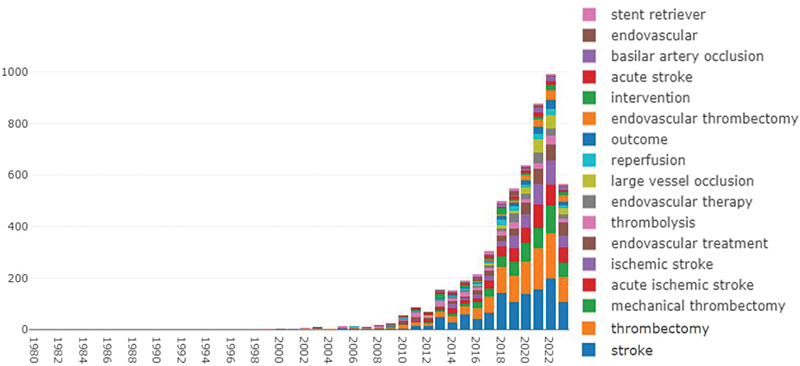
The diagram of the annual composition of high-frequency keywords.

**Figure 5:**
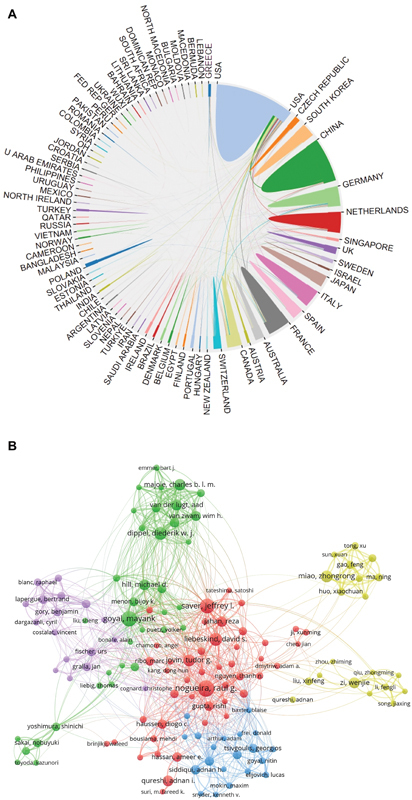
The network diagram of cooperation relationship among countries and the network diagram of the cooperation relationship among authors. ( **A** ) Cooperation among countries. ( **B** ) Cooperation among individuals.

- The top 10 most-cited articles;
- The top 10 most-cited institutions;
- The top 10 most-cited journals;
- The top 10 most-productive authors;
- The number of articles published per year;
- The number of articles published per country;
- The network diagram of high-frequency keywords;
- The diagram illustrating the composition of high-frequency keywords annually;
- The network diagram of cooperation relationships among countries; and
- The network diagram of cooperation relationships among authors.

To ensure the reliability of the research results, data collation and analysis were completed by two neurointerventional experts and a statistical expert.

## RESULTS

### General situation

A total of 5,576 articles were retrieved. The top 100 most-cited ones were selected to analyze the research hotspots, and Table 2 presents the top 10 articles with the highest number of citations. Among them, the most-cited article is the one by Berkhemer et al., 6 titled “A Randomized Trial of Intraarterial Treatment for Acute Ischemic Stroke,” and published in 2015, which accumulated 4,283 citations.

### The number of articles published per year

As depicted in Figure 2A , an obvious increase in the number of articles occurred after 2010, indicating a growing interest in the field. Particularly notable increases were observed in 2015 and 2019. Furthermore, Figure 2A illustrates that the United States consistently contributed the highest proportion of annual publications compared to other countries. Notably, the number of articles published by Chinese researchers has shown a remarkable rise since 2015.

### The number of articles published per country

Figure 2B presents a heat map showcasing the distribution of articles published per country.

### Keyword analysis

Figures 4^5^ reveal the analysis of high-frequency keywords, including terms such as large vessel occlusion, reperfusion, outcome, and basilar artery occlusion, among others.

### The most-cited institutions

Table 3 outlines the top 10 most-cited institutions, with the University of Calgary ranking first. Notably, among these institutions, the Bellvitge University Hospital (Hospital Universitari de Bellvitge), in Barcelona, Spain, exhibited the highest average number of citations per article, with an average of 203 citations.

### The most-productive authors

Table 5 describes the top 10 most-productive authors, with Raul G. Nogueira leading the list, with a total of 136 published articles.

### The most-cited journals

Table 4 presents the top 10 most-cited journals in the field. *The New England Journal of Medicine* ranked first, with 5,631 citations. Notably, *The Lancet* had an average of 151 citations per article, surpassing other journals in terms of citation impact. Among the top 10 most-cited journals, *Stroke* had the highest number of articles, totaling 903 publications.

### Cooperation relationship among countries/authors

Figure 5A illustrates the cooperation relationship among countries, demonstrating that the United States has the closest collaborative ties with other nations. In Figure 5B , Raul G. Nogueira and Jeffrey Saver, from the United States, as well as Mayank Goyal, from Canada, emerged as the most popular collaborators within the field.

## DISCUSSION

Mechanical thrombectomy plays a pivotal role in AIS endovascular treatment. Stent retriever thrombectomy and aspiration thrombectomy are the primary techniques adopted in mechanical thrombectomy. Unless otherwise specified, the term *endovascular treatment for AIS* generally refers to mechanical thrombectomy. It should be acknowledged that mechanical thrombectomy, while effective, can also pose risks of complications such as hemorrhagic transformation, iatrogenic embolization, and vasospasm.

In the present analysis, we explored various data characteristics pertaining to AIS endovascular treatment, including publication date, countries involved, contributing authors, prominent keywords, collaborative relationships, and more. By scrutinizing the full text of the top 100 most-cited articles and identifying high-frequency keywords, we have identified several notable research hotspots within this field.

### A milestone in the field of endovascular treatment for AIS

The most-cited article reported the results of the Multicenter Randomized Clinical Trial of Endovascular Treatment of Acute Ischemic Stroke in the Netherlands (MR CLEAN). 6 The MR CLEAN trial stood out as the first large-scale randomized controlled study to conclusively demonstrate the effectiveness of the endovascular treatment for AIS. Given its meaningful impact, this article 6 garnered significant attention and is widely regarded as a landmark achievement in the field.

Furthermore, in 2015, four prospective studies 4 1 7 5 with positive results further contributed to the advancement of the endovascular treatment for AIS. These studies included the Endovascular Treatment for Small Core and Anterior Circulation Proximal Occlusion with Emphasis on Minimizing CT to Recanalization times ESCAPE) trial, 4 the Solitaire with the Intention for Thrombectomy as Primary Endovascular Treatment for Acute Ischemic Stroke (SWIFT PRIME) trial, 1 the Endovascular Revascularization with Solitaire Device versus Best Medical Therapy in Anterior Circulation Stroke within 8 Hours (REVASCAT) trial, 7 and the Extending the Time for Thrombolysis in Emergency Neurological Deficits with Intra-arterial Therapy (EXTEND-IA) trial. 5 A meta-analysis incorporating these 5 studies demonstrated that the endovascular treatment for AIS could improve functional prognosis 90 days after discharge. 8

### Research hotspots

The endovascular treatment for AIS has been established as an effective approach to improve patient prognosis following mechanical thrombectomy. However, several controversial topics persist within this field. Through the analysis of total and annual high-frequency keywords ( Figures 3 , 4 ) and the top 100 most-cited articles, we have identified the following research hotspots.

#### *Core infarction/ischemic penumbra*

In patients undergoing mechanical thrombectomy, the size of the core infarction is correlated with prognosis. Larger core infarctions are associated with worse outcome and a higher risk of complications, such as hemorrhagic transformation. The SWIFT PRIME 1 and the EXTEND-IA 5 trials used core infarct volumes > 50 mL and > 70 mL as contraindications for mechanical thrombectomy respectively.

Besides, the ischemic penumbra is the main target for AIS treatment. The study titled A Multicenter Randomized Controlled Trial of Endovascular Therapy following Imaging Evaluation for Ischemic Stroke (DEFUSE 3) used an ischemic/infarct ratio ≥ 1.8 and a mismatch volume between ischemia and infarct > 15 mL as indications for mechanical thrombectomy. 9

#### *Collateral circulation*

Collateral circulation plays a crucial role in compensating for the ischemic region and significantly influences the prognosis of patients undergoing mechanical thrombectomy. The collateral flow grading system developed by the American Society of Interventional and Therapeutic Neuroradiology/Society of Interventional Radiology (ASITN/SIR) is widely adopted in international clinical studies to classify collateral circulation. 10

Despite that, it is important to note that the cerebral angiography evaluation of the collateral circulation has certain limitations. Firstly, it lacks the ability to provide a quantitative assessment of the local cerebral blood flow. This hinders an exhaustive understanding of the perfusion status within the affected brain regions. Secondly, cerebral angiography involves the use of a high-pressure injector and a specific amount of contrast agent. Variations in the dosage and pressure of contrast agents can exert an influence on the visualization of distal vessels, potentially affecting the accuracy of collateral circulation assessment. These limitations underscore the need for alternative methods to accurately evaluate collateral circulation. We should focus on developing non-invasive techniques that enable a quantitative assessment of the cerebral blood flow and improve the visualization of distal and collateral vessels.

#### *Time window versus “tissue window”*

The prevailing belief was that early endovascular treatment within the designated time window yields the most favorable outcomes, while the effectiveness of treatment diminishes when performed beyond this window. However, with advancements in research, there is a growing recognition that there are also treatment opportunities for patients beyond the time window. In other words, for patients beyond the time window, we can use imaging to evaluate the presence of reversible ischemic penumbra tissue to expand the indication for mechanical thrombectomy. This assessment is usually performed by checking for the existence of a mismatch between the ischemic penumbra and the core infarct area. This “tissue window” concept has begun to be recognized by numerous doctors.

For instance, the MR-selected Patients with Stroke of Unknown Onset (MR WITNESS) trial and the MRI-guided Thrombolysis for Stroke with Unknown Time of Onset (WAKE-UP) trial have demonstrated the efficacy of using diffusion-weighted magnetic resonance imaging (DW-MRI) and fluid-attenuated inversion recovery (FLAIR) in the identification of a mismatch and guide the treatment for AIS. 11 Consequently, it becomes essential to assess the “tissue window” when performing mechanical thrombectomy on patients who fall outside the traditional 6-hour time window. 12

In the DWI or CTP Assessment with Clinical Mismatch in the Triage of Wake-up and Late-presenting Strokes Undergoing Neuro-intervention with Trevo (DAWN) trial, endovascular treatment with the Trevo device (Stryker, Portage, MI, United States) was performed in patients with wake-up and late-presenting strokes who had an imaging and clinical mismatch. The results indicated that the time window for thrombectomy can be extended from 6 hours to 24 hours for patients with acute anterior circulation large artery occlusion. At the same time, 49% of the patients achieved a good prognosis under the guidance of tissue window, while only 13% of the patients achieved a good prognosis under the guidance of the traditional time window. 13

#### *Stent retriever thrombectomy versus aspiration thrombectomy*

Stent retriever thrombectomy and aspiration thrombectomy are the two main methods adopted for thrombectomy procedures. A meta-analysis reported that aspiration thrombectomy, in comparison to stent retriever thrombectomy, yields higher reperfusion rates and lower risks of complications. 14 On the contrary, some differing opinions exist, suggesting that there may not be a significant difference between these two methods. 15

In the clinical practice, it is important to consider various factors such as the surgeon's experience and understanding of material properties and characteristics of vascular anatomy when selecting the appropriate treatment method. Complex situations, such as high thrombus burden, bifurcation lesions, involvement of distal branch vessels, or tough thrombus, may require combining both methods to achieve complete reperfusion (grade 3 on the modified Thrombolysis in Cerebral Infarction [mTICI] score).

#### *Bridging therapy*

Bridging therapy can be categorized into two types: direct bridging treatment and salvage bridging treatment. In recent years, six large clinical trials 16 17 18 19 20 21 focused on the issue of “direct versus bridging therapy”, namely the Direct Intra-arterial Ihrombectomy in Order to Revascularize AIS Patients with Large Vessel Occlusion Efficiently in Chinese Tertiary Hospitals: A Multicenter Randomized Clinical Trial (DIRECT-MT), 16 the Endovascular Treatment Alone versus Intravenous Alteplase plus Endovascular Treatment on Functional Independence in Patients with AIS (DEVT) trial, 17 the Mechanical Thrombectomy without versus with Intravenous Thrombolysis on Functional Outcome among Patients with AIS (SKIP) trial, 18 the Admission Blood Pressure and Clinical Outcomes in Patients with AIS Treated with Intravenous Alteplase and Endovascular Treatment versus Endovascular Treatment Alone (MR CLEAN-NO IV) trial, 19 the Solitaire with the Intention for Thrombectomy plus Intravenous t-PA versus Direct Solitaire Stent-retriever Thrombectomy in Acute Anterior Circulation Stroke SWIFT DIRECT) trial, 20 and the Randomized Controlled Trial of Direct Endovascular Clot Retrieval versus Standard Bridging Therapy (DIRECT-SAFE). 21 The DIRECT-MT 16 and DEVT 17 trials showed the noninferiority of direct thrombectomy to bridging therapy, but not the other four studies. 18 19 20 21 However, there were different views suggesting that bridging therapy may reduce mortality. 22

In order to better understand the clinical application of direct thrombectomy and bridging therapy, we have listed the latest treatment guidelines from various countries. In the 2019 AHA/ASA guidelines for the management of AIS, intravenous alteplase (also called recombinant tissue plasminogen activator, rTPA) thrombolysis should be chosen first, even if mechanical thrombectomy has been considered. Patients undergoing mechanical thrombectomy should not wait for the therapeutic effect of rTPA thrombolysis. According to the 2021 Japanese guidelines for AIS, 24 intravenous rTPA is strongly recommended for patients without contraindications within the time window. Endovascular treatment should be performed as soon as possible after the patient's arrival. 24 The 2023 National Clinical Guideline for Stroke for the United Kingdom and Ireland (available at www.strokeguideline.org ) recommended that AIS patients who are eligible for mechanical thrombectomy should be given intravenous thrombolysis as soon as possible before thrombectomy, within 4.5 hours of symptom onset (unless there are contraindications).

Indeed, it is crucial to emphasize that bridging therapy should be implemented without causing any delay in the initiation of mechanical thrombectomy. Continued research efforts and evidence from real-world practice will contribute to enhance our understanding of the optimal selection.

#### *Basilar artery occlusion*

Basilar artery occlusion can have severe clinical consequences, necessitating effective treatment strategies. While the efficacy of endovascular treatment for large vessel occlusion in acute anterior circulation is well-established, its effectiveness in acute basilar artery occlusion remains unclear. Studies 26 27 have reported that the proportion of patients achieving a good prognosis (score on the Modified Rankin Scale, mRS, ≤ 2) following endovascular treatment for basilar artery occlusion is of approximately 30%.

Two randomized controlled trials, the Basilar Artery Occlusion Endovascular Intervention versus Standard Medical Treatment (BEST) 28 and the Basilar Artery International Cooperation Study (BASICS), 29 have demonstrated better outcomes compared to standard medical treatment. However, the observed difference did not reach statistical significance. In 2022, two trials conducted in China, Endovascular Treatment of Acute Basilar Artery Occlusion (ATTENTION) 30 and Basilar Artery Occlusion in China (BAOCHE), 31 revealed that endovascular thrombectomy performed within 24 hours of symptom onset could reduce the rate of mortality, particularly in patients with moderate-to-severe symptoms. The risk of intracranial hemorrhage complications in patients undergoing thrombectomy for basilar artery occlusion appears to be similar to that observed in cases of anterior circulation occlusion. While these findings provide valuable insights, further research is needed to establish optimal treatment strategies and clarify the role of endovascular treatment in acute basilar artery occlusion.

#### *Choice of anesthesia*

The potential association between general anesthesia and unfavorable prognosis in patients undergoing thrombectomy remains a topic of ongoing debate. 32 33 34 Nevertheless, the timely achievement of recanalization and meticulous control of blood pressure are widely acknowledged as being more beneficial than the specific choice of anesthesia. In cases in which the patient can cooperate during the thrombectomy procedure, local anesthesia is usually preferred because it can reduce the wait time for surgical recanalization. Conversely, patients presenting with agitation may benefit from intravenous anesthesia. 35 It is worth noting that general anesthesia with tracheal intubation is the first choice for patients with severe airway obstruction, because it can effectively reduce the risk of choking and hypoxia.

### Big data analysis of bibliometric characteristics

#### *Publication date ( Figure 2A )*

The Mechanical Embolus Removal in Cerebral Ischemia (MERCI) thrombectomy device (Concentric Medical, Inc., Mountain View, CA, United States) gained approval from the US Food and Drug Administration (FDA) for mechanical thrombectomy in 2004. 36 Subsequently, around 2010, the second generation of thrombectomy devices, such as the PENUMBRA thrombectomy catheter, and the third generation of thrombectomy devices, such as the Solitaire stent, were introduced. 37

The use of these new instruments made mechanical thrombectomy safer and more effective. Therefore, more and more researchers began to explore the clinical application of mechanical thrombectomy, resulting in a significant increase in the number of studies on endovascular therapy for AIS after 2010.

#### *The countries with the highest number of published articles ( Figure 2B ) and the most-cited institutions ( Table 3 )*

Figure 2B highlights that the United States and Europe hold a prominent position in terms of the quantity of published articles concerning endovascular treatment for AIS. Similarly, Table 3 indicates that the institutions most frequently cited predominantly originate from the United States and European countries.

Several factors contribute to this phenomenon. First, the United States and Europe have advanced medical research equipment and technology. Moreover, these regions have established comprehensive medical services and refined insurance systems. Research institutions and universities within these areas exhibit robust scientific research capabilities, often backed by adequate financial support. Consequently, an environment conducive to cerebrovascular physicians and researchers has been provided, facilitating the development and advancement of endovascular treatment research for AIS.

#### *The most-cited journals ( Table 4 )*

*The New England Journal of Medicine* was the journal with the highest citation count, underscoring its substantial influence within the field. Moreover, *The Lancet* exhibited the highest number of citations per article, suggesting a stringent standard for research quality and rigorous peer review process. This elevated metric may be indicative of the journal's commitment to publishing impactful studies. Additionally, the *Stroke* journal boasts the largest number of published articles, reflecting its status as the preferred outlet for submission among cerebrovascular physicians. Its widespread popularity as a platform to disseminate research findings in this domain further solidifies its significance within the scientific community.

#### *Cooperation among countries ( Figure 5A )*

According to Figure 5A , the United States exhibits the closest cooperation with other countries in the field of mechanical thrombectomy. This observation can be attributed to the following factors: the United States has more research resources and a mature interdisciplinary cooperation mechanism. These rich scientific resources provide a solid foundation to conduct high-quality research in the field of mechanical thrombectomy. This interdisciplinary cooperation promotes the collaborative interaction of researchers in different fields, as well as the rapid development of mechanical thrombectomy research.

#### *Cooperation among individuals ( Figure 5B )*

Based on the findings depicted in Figure 5B , it is evident that Raul G. Nogueira and Jeffrey Saver, from the United States, along with Mayank Goyal, from Canada, are prominent collaborators within the field. Their notable popularity can be attributed to their leadership roles or active involvement in a multitude of high-quality research studies. 38 39 40 41 42 43 44

#### *The most productive author ( Table 5 )*

In the present study, Raul G. Nogueira emerged as the most prolific author, with a remarkable publication record comprising 136 articles. He participated in several classic experiments, and these clinical trials provide an important reference for the formulation of clinical guidelines. 1 13

### Limitations to the study

- Limitations regarding data sources: relying on the published literature as the main data source may ignore the unpublished research results;
- Limitation in the number of citations: using the number of citations as a standard to measure scientific impact has certain limitations, because the number of citations is affected by domain-specific citation habits, self-citation, and other factors;
- Domain specificity: there may be differences in the citation habits and methods of dissemination of literature in different fields, so the results of bibliometrics may be affected by domain specificity;
- Time delay: because bibliometric research needs to be based on published literature, there may be time delay, which cannot reflect the latest research progress and results in a timely manner.

In conclusion, we found that the reports on the endovascular treatment for AIS gradually increased after 2010. Among them, Raul G. Nogueira was the most productive author. *The New England Journal of Medicine* was cited the most and had the greatest impact. The MR CLEAN trial study has been cited most and was a landmark study. There are many interesting studies on endovascular treatment for AIS, such as ischemic penumbra, collateral circulation, bridging therapy etc.
